# Living arrangements of the elderly and the sociodemographic and health determinants: a longitudinal study**^1^**

**DOI:** 10.1590/1518-8345.0668.2737

**Published:** 2016-08-08

**Authors:** Alisson Fernandes Bolina, Darlene Mara dos Santos Tavares

**Affiliations:** 2 Doctoral Student, Escola de Enfermagem de Ribeirão Preto, Universidade de São Paulo, PAHO/WHO Collaborating Centre for Nursing Research Development, Ribeirão Preto, SP, Brazil.; 3 PhD, Associate Professor, Departamento de Enfermagem em Educação e Saúde Comunitária, Instituto de Ciências da Saúde, Universidade Federal do Triângulo Mineiro, Uberaba, MG, Brazil.

**Keywords:** Aged, Family, Aging

## Abstract

**Objectives:**

to describe the sociodemographic characteristics and the number of morbidities in the elderly, according to the dynamics of living arrangements and evaluate the sociodemographic and health determinants of the living arrangements.

**Methods:**

this is a household longitudinal survey (2005-2012), carried out with 623 elderly people. Descriptive statistical analysis and multinomial logistic regression were performed (p<0.05).

**Results:**

there was predominance of elderly living alone, accompanied and with change in the living arrangements, females, age range between 60├ 70 years, 1├ 4 years of study and with income between 1├┤ 3 minimum wages. During the development of this research, it was identified an increase in the incidence of elderly with 1├┤3 minimum wages. The number of morbidities increased in the three groups throughout the study, with the highest rates observed among the elderly with change in the dynamics of living arrangements. It was found that elderly men showed less chance of living alone (p=0.007) and having change in the living arrangements compared to women (p = 0.005). Incomes less than a minimum wage decreased the chances of change in the living arrangements compared to incomes above three salaries (p=0.034).

**Conclusion:**

the determining factors of the living arrangements were sex and income, and the variables functional capacity and number of morbidities were not associated with the outcome analyzed.

## Introduction

Population aging has caused changes in the structure of families^1^. There are several concepts that characterize the family according to the different approaches adopted in the areas of science. Anthropology defines it as the "group of people linked by affective bonds built on a basis of consanguinity and alliance"^2^ and the sociological, demographic and economic studies limit the concept to physical spaces by considering the family as a group of individuals residing in the same household^3^. 

Housing can be understood as "a structurally independent and separate location that is intended to serve as residence for one or more people, or that is used for such purposes"^4^. Thus, the way in which an individual - or a group of individuals - is settled down in that physical space constitutes the living arrangements^3^ - it is the concept adopted in this study. 

The living arrangements are complex and have diversified compositions^2^. The heterogeneity of these arrangements could be a reflection of the different determinants influencing their structure^5^. In general, it is highlighted their cultural, demographic, socioeconomic and health characteristics^5^ ^-^ ^6^.

Despite the importance of understanding the family arrangement for implementation of social and health policies more assertive to the needs of the elderly and their family^7^, publications on the theme are still scarce. To date, no longitudinal study evaluating the determinants of the living arrangements in Brazilian elderly was found. 

In Brazil, although cohabitation remains high among the elderly population, it has been observed throughout the years, an increasing number of individuals who live in single person households^5^. The single person households may represent an achievement of aging to the extent that this group experiences more privacy and independence in the course of aging. However, this type of living arrangement can also shelter the elderly who need assistance in daily life, especially those with health and family problems or poor living conditions^5^ ^-^ ^7^. Hence, knowing the characteristics of the living arrangements of the elderly can provide subsidies for the detection of vulnerable groups that need to be prioritized in health care by primary care professionals, including nurses.

The objectives of this study were: to describe the sociodemographic characteristics and the number of morbidities of the elderly, according to the dynamics of living arrangements; and evaluate the sociodemographic and health determinants of this composition.

## Methods

This is a household quantitative longitudinal study conducted with elderly people in the municipality of Uberaba - MG, in the period from 2005 to 2012. 

The study population consisted of elderly people residing in the municipality mentioned. In 2005, an initial sample was calculated (2892 elderly persons), considering a confidence interval of 95%, a power of 80% and a margin of error of 4.0% for interval estimates, and an estimated proportion of π = 0.5 for the proportions of interest. 

**Figure 1:**
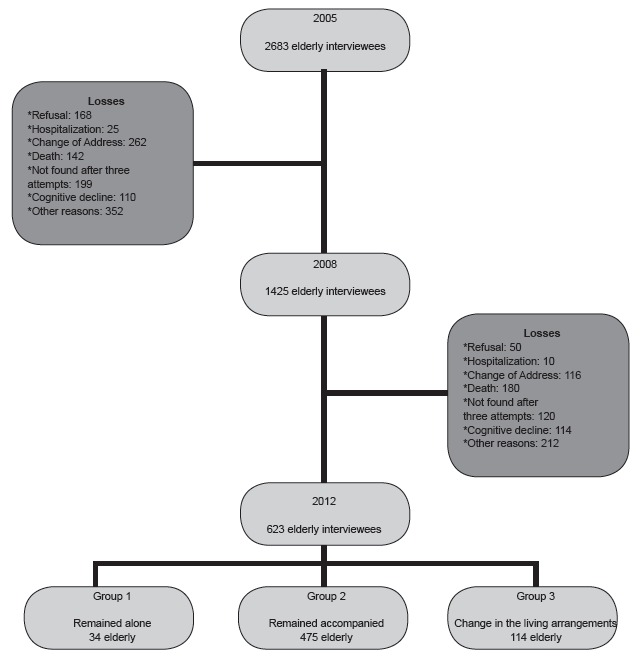
Algorithm for the final sample description.

Thereby, information of 2,683 elderly people was obtained, as a result of refusals. Later, in 2008 and 2012, elderly were located and interviewed again. In 2008, interviews were conducted with 1,425 individuals and 2012, with 623. The decrease in the number of interviewees, throughout follow-up, occurred due to several reasons such as: elderly be hospitalized; have changed address; have died; or could not be found after three attempts of visit by the interviewer, among others, as shown in Figure 1.

Inclusion criteria were: be 60 years of age or older; not to present cognitive decline; live in the urban area in the municipality of Uberaba - MG; be interviewed in the three occasions (2005, 2008 and 2012); and agree to participate. In this way, the participants of this research totaled 623 elderly people.

The elderly enrolled in this study were divided into three groups according to the dynamics of living arrangements during follow-up: group 1 (Alone) - elderly people who remained alone during follow-up; group 2 (Accompanied) - elderly people who lived accompanied during follow-up; and group 3 (Change in the living arrangements) - elderly people with change in the living arrangements during follow-up (Figure 1).

Data collection was performed at the residence of the elderly, using the proportionate stratified sampling method for their selection and considering the various neighborhoods as strata. 

The Mini-Mental State Examination (MMSE) was used in the cognitive assessment. The short version of MMSE, validated by researchers of the Health, Welfare and Aging (SABE), was used in 2005 and 2008^8^. In this version, the cutoff point was set as 12/13 and scoring less than 12^8^ indicated cognitive impairment. In 2012, it was used the MMSE version translated and validated in Brazil^9^, which is composed by questions regarding to orientation, immediate and evocation memory, concentration, calculation, language and spatial domain. That year, it was considered that score could vary and the cut-off point was set according to the education level of the elderly: 13 for illiterates, 18 for 1 to 11 years of study and 26 for more than 11 years of study ^9^. This change was implemented because the researchers considered the validated instrument and the cutoff score^9^ more suitable for the case.

To characterize the sociodemographic data, the morbidity and functional capacity, it was used part of the questionnaire Older Americans Resources and Services (OARS), developed by Duke University (1978) and adapted in Brazil by Ramos (1987), with the designation of Brazilian version of Older Multidimensional Functional Assessment Questionnaire (BOMFAQ)^10^.

Regarding the morbidities assessment, the questionnaire contains 26 items investigating whether the individual presents morbidity^10^. The number of morbidities reported by the elderly was accounted for data analysis. 

Regarding the functional capacity assessment, the questionnaire comprises 12 activities of daily living, by which the elderly tells the difficulty level to perform self-care activities (unable to do, little difficulty, much difficulty and no difficulty)^10^. For statistical analysis, it was calculated the score of functional capacity that corresponds to the sum of the scoring of activities of daily living: 1 (Unable to do), 2 (Little difficulty), 3 (Much difficulty) and 4 (No difficulty). Thus, the overall score of functional capacity ranges from 12 to 48, and the highest scores are associated with better functional capacity.

The variables investigated were: gender (male and female); age group (60|-70, 70|-80 and 80 years or over); educational level, in years of study (No schooling, 1├4 and > 5); living arrangements (Alone and other arrangements); individual monthly income, in minimum wages (< 1, 1|┤ 3 and > 4); number of self-reported morbidities; and score of functional capacity. 

For data analysis, spreadsheets were built in Excel(r). The data collected in each period were processed in a personal computer, with double entry, to verify the consistency between the two databases. For data analysis, database was exported to the *Statistical Package for Social Science* software (SPSS), version 17.0. 

Descriptive statistical analysis was performed by means of distribution of absolute and percentage frequencies. Multivariate analysis through multinomial logistic regression was used to verify the determinants of the living arrangements. In relation to the predictive variables such as gender, age and education, it was considered the data regarding 2005; whereas for the variables income, number of morbidities and score of functional capacity, the average of the three periods was calculated. A significance level of p < 0,05 for adopted for the multivariate model.

The projects were approved by the Ethics Committee on Human Research of the Federal University of Triângulo Mineiro, under Protocol number 553 in 2005, number 897 in 2008 and number 265 in 2012. The elderly were contacted at home, when the objectives and other relevant information were presented. The interview was conducted after the participants have approved and assigned the Informed Consent form.

## Results

The study population consisted of 623 elderly people interviewed during the study, divided into three groups according to the dynamics of living arrangements: 34 (5.5%) remained alone, 475 (76.2%) lived accompanied and 114 (18, 3%) presented change in the living arrangements. 

In all three groups, most elderly were women, and those who lived alone accounted for the highest percentage (82.4%); unlike observed among men, among which predominated (35.2%) those who lived accompanied (Table 1).

**Table 1:** Distribution of sex, age group and education level among elderly, according to the dynamics of living arrangements in the baseline of follow-up (2005). Uberaba, MG, Brazil, 2005.

Variables		Dynamics of Living Arrangements							
		Alone			Accompanied			Change	
		n	%		n	%		n	%
Sex									
	Female	28	82.4		308	64.8		87	76.3
	Male	6	17.6		167	35.2		27	23.7
Age group									
	60├ 70 years	19	57.6		291	61.3		68	59.3
	70├ 80 years	11	33.3		155	32.6		38	33.6
	80 or over	3	9.1		29	6.1		8	7.1
Education (Years of study)*									
	0	6	17.6		104	21.9		27	23.9
	1├ 4 years	23	67.6		260	54.9		62	54.9
	>5 years	5	14.7		110	23.2		24	21.2

* Missing data: Group change (1)

In 2005, most of this population were aged between 60├ 70 years, and in this age group, the highest percentage corresponded to people living accompanied (61.3%). Among those aged between 70├ 80 years, those with changes in the living arrangements predominated (33.6%). Elderly aged 80 years or over represented the highest percentage among those living alone (9.1%) (Table 1).

Regarding schooling, there was a predominance of elderly with 1├ 4 years of study, followed by those with no schooling, in the three groups. Among those with no schooling, there was a prevalence of individuals who experienced changes in the living arrangements (23.9%); among those with 1├ 4 years of study, those living alone (67.6%); and among those with more than five years of study, the ones living accompanied (23.2%) (Table 1). The distribution of the educational level did not change during the study.

Table 1 shows the distribution of gender, age and education level of the elderly according to the living arrangements.

Most elderly who lived alone, accompanied and with change in the living arrangements had 1├┤ 3 minimum wages in 2005, 2008 and 2012 - with an increase in the distribution of individuals of this income bracket during follow-up. In the course of study period, there was an increase in the percentage of participants with 1├┤3 minimum wages in the three groups, and the elderly who lived alone represented the highest percentage compared to the others (Table 2). 

**Table 2:** Distribution of individual income bracket and number of morbidities among elderly, according to the dynamics of living arrangements in the three follow-up periods, Uberaba, MG, Brazil, 2005, 2008 and 2012.

Year	Variables		Dynamics of Living Arrangements							
			Alone			Accompanied			Change	
			n	%		n	%		n	%
	Income^*^									
2005^†^		<1	4	11.8		107	22.8		17	15.2
		1├┤3	27	79.4		302	64.2		85	75.9
		>3	3	8.8		61	13.0		10	8.9
2008^†^		<1	2	5.9		63	13.4		10	8.8
		1├┤3	27	79.4		368	78.5		96	85.0
		>3	5	14.7		38	8.1		7	6.2
2012^†^		<1	0	0		50	10.5		4	3.6
		1├┤3	31	91.2		382	80.9		99	86.8
		>3	3	8.8		40	8.5		11	9.6
	Number of morbidities									
2005		0	0	0		1	0.2		0	0
		1├┤2	16	47.1		221	46.7		59	51.8
		3├┤4	6	17.6		87	18.3		18	15.8
		>4	12	35.3		166	34.9		37	32.5
2008		0	1	2.9		9	1.9		1	0.9
		1├┤2	6	17.6		52	10.9		9	7.9
		3├┤4	4	11.8		111	23.4		23	20.2
		>4	23	67.6		303	63.8		81	71.1
2012		0	3	8.8		9	1.9		1	0.9
		1├┤2	3	8.8		71	14.9		20	17.5
		3├┤4	11	32.4		122	25.7		20	17.5
		>4	17	50.0		273	57.5		73	64.0

*Minimum wage during data collection period: 2005 (R$ 300.00); 2008 (R$ 415.00) and 2012 (R$ 622.00)(11).

†Missing data:

2005-group accompanied (5) and change (2).

2008-group accompanied (6) and change (1).

2012-group accompanied (3).

Concerning the number of morbidities in 2005, there was a predominance of elderly with 1├┤2 diseases in the three groups, with the highest percentages observed among those who experienced change in the living arrangements (51.8%). In subsequent follow-ups, most individuals had more than four morbidities, and the elderly who experienced change in the living arrangements represented the highest percentages (71.7%) in 2008 and (64.0%) in 2012 (Table 2). 

Table 2 below shows the distribution of age and number of morbidities among elderly, according to the dynamics of living arrangements.

Regarding the determinants of the living arrangements, it was found that elderly men had approximately 75% less chance to live alone (*p*=0.007) and 51% less chance to change the living arrangements compared to women (*p*=0.005) (Table 3).

**Table 3:** Regression model of the determinants of the living arrangements of the elderly, Uberaba, MG, Brazil, 2005, 2008 and 2012

Variables		Living Arrangements							
		Alone*			Change*	
		OR^†^ (95%CI)^‡^	p		OR† (95%CI)^‡^	p
Sex						
	Male	0.249 (0.090-0.688)	0.007		0.490 (0.297-0.808)	0.005
	Female	-	-		-	-
Age group						
	60├ 70	0.471 (0.122-1.812)	0.273		0.796 (0.338-1.876)	0.602
	70├ 80	0.462 (0.114-1.868)	0.279		0.749 (0.308-1.820)	0.524
	80 or over	-	-		-	-
Education						
	0	0.924 (0.213-4.015)	0.917		1.390 (0.723-2.671)	0.324
	1├ 4	2.157 (0.819-5.682)	0.120		1.038 (0.614-1.754)	0.889
	>5	-	-		-	-
Income						
	<1	0.222 (0.032-1.559)	0.130		0.282 (0.088-0.908)	0.034
	1├┤3	0.808 (0.217-3.008)	0.750		0.938 (0.430-2.048)	0.873
	>3	-	-		-	-
Number of morbidities		1.024 (0.829-1.265)	0.827		1.045 (0.926-1.179)	0.478
Functional capacity		1.162 (0.965-1.399)	0.114		1.078 (0.983-1.182)	0.108

*Reference category: Accompanied

†Odds Ratio

‡Confidence interval

The income bracket was another determinant of change in the living arrangements. To earn less than a minimum wage decreases by 72%, approximately, the chances of change in the arrangement in relation to individuals with more than three minimum wages (*p* = 0.034) (Table 3).

 It is emphasized that the functional capacity and the number of morbidities were not associated with living alone and the change in the living arrangements (Table 3).

The determining factors of the living arrangements of the elderly are listed in Table 3, as shown below.

## Discussion

In this investigation, the predominance of elderly women living alone was, probably, due to their greater longevity^5^, which increases their chances of becoming widows^12^. In addition, if men become widowed, they often marry again, unlike women^13^. Investigations carried out in China and Ribeirão Preto - SP also found a higher percentage of elderly women in single person households, (67.2%)^14^ and (71.4%)^15^, respectively. 

In a study performed in the United States, it was also observed the predominance of younger elderly living accompanied. Most individuals aged between 65 and 69 lived with someone else (73.0%)^16^. However, conflicting results have been found in other studies conducted at international level^17^ ^-^ ^19^. The differences in the results of these studies may be explained by the dual relationship between age and living arrangements. The course of aging can make the elderly live alone because of the loss of their spouses, but it also increases the chances of the elderly cohabit as a result of a greater physical dependence^5^ ^-^ ^6^. 

Some authors suggest that physical dependence in the elderly is intensified from 80 years of age^15^. In this way, the fact that a higher percentage of octogenarian individuals remained living alone during the follow-up demonstrates, on the one hand, a satisfactory finding, since it is inferred that they are experiencing a successful aging process. However, on the other hand, this is a matter of concern, since these elderly may face some impasses in the use of health services, and have trouble to perform everyday tasks, which are exacerbated in the absence of a family member. 

Regarding schooling, the results of this study differ from others conducted in China^18^. This study demonstrates that higher levels of education increase the proportion of elderly living alone^18^. This may be due to the choice of a lifestyle with more privacy and independence. In contrast, elderly with lower level of education have larger families^18^, which may favor cohabitation. Thus, schooling interferes either directly in the living arrangements, by influencing the perception of the elderly about the best option of living, and indirectly, by having an impact on other aspects, such as the number of children. 

In contrast, other studies have found that elderly with lower level of education lived alone^14^ ^,^ ^16^. It is believed that there are differences in the relation between education level and living arrangements, according to the cultural characteristics of each region. In some Latin American countries such as Brazil, the proportion of elderly living accompanied, specifically with their sons, increases among individuals with higher levels of education^18^, corroborating the present study.

There was a predominance of a higher income among elderly living alone, which confirmed a study conducted in São Paulo^6^. However, a survey performed at international level showed a predominance of elderly with lower incomes living in this type of living arrangement^16^. Elderly with higher incomes tend to live alone as way to preserve their privacy and independence^6^. In addition, the economic availability can offer a greater purchasing power, providing easier access to health services, as well as a more adequate social cover^6^ ^,^ ^12^ ^,^ ^20^ - essential aspects to live on their own. In contrast, many elderly with higher incomes reside with their families because of the economic needs of their sons, who make use of their housing and financial resources^7^.

The high incidence of diseases among elderly with change in the living arrangements corroborates a study carried out in the municipality of Campinas, which revealed that the number of morbidities was among the determining factors of the type of family configuration of the elderly^7^. This finding may be related to the need for physical aid, causing many to live with their families and not in single person households^15^. 

It is important to point out that cohabitation, by itself, does not mean support in times of difficulty. Several theories on the family system and types of living arrangements, historically emphasize the protective role of the family on the living conditions of the elderly, especially in societies whose assistance policies for this population are still not consolidated^21^. However, the results of recent studies at international level are contradictory; some corroborate these hypotheses^16^ ^-^ ^17^ ^,^ ^22^, whereas others suggest a contrary perspective^1^ ^,^ ^14^ ^,^ ^21^, which indicates the need of further analysis of the situation by means of research.

In the analysis of the determinants of the living arrangements, the association between female gender and living alone differs from a study conducted with Japanese elderly, which revealed a higher proportion of men living alone compared to women (*p* = 0.049)^22^. Other research conducted with Indian elderly also found divergent results, revealing that sex had no statistically significant effect on the other variables (p=0.959)^18^. On the other hand, the findings of international studies corroborate the present investigation^14^ ^,^ ^17^ ^,^ ^19^. These studies found a higher proportion of elderly women living alone compared to men^14^ ^,^ ^17^ ^,^ ^19^. 

The female sex was also associated with higher chances of change in the living arrangements in a study of elderly in the state of Rio Grande do Sul^12^. This result is not surprising, considering that it is known that women have longer life expectancy and therefore higher chances of becoming widows^12^. The longer life expectancy in women has been one of the concerns highlighted by the World Health Organization, since the elder elderly women may have more disabilities as well as other multiple health problems^23^. Added to this, with the course of aging, women are more vulnerable to health problems, social isolation and emotional disorders^24^. Thus, social policies should consider the situation of elderly women, especially the most vulnerable groups such as those living in single person households^17^.

In this study, income was another determining factor of the living arrangements. High income can function as facilitator of independent living arrangements, since it results in a greater purchasing power for the purchase of consumer goods, as well as more adequate social and health coverages^6^ ^,^ ^12^ ^,^ ^20^, proving that elderly with high socioeconomic level generally prefer a more independent lifestyle^6^. These aspects possibly contribute to the establishment of single person households among elderly population. In opposition to these arguments, economic independence has been considered an aspect that favors cohabitation because family members often live with the elderly in an attempt to take advantage of the available financial resources^18^. Despite this, a study carried out in Ribeirão Preto - SP found that income was not among the reasons that influenced the living arrangements of the elderly^15^. 

 It must be considered that studies on the determinants of the living arrangements generally assess the outcome punctually - living alone or accompanied - without evaluating the dynamics of living arrangements and their follow-up. This hindered the discussion of our findings, but at the same time raises the need to expand the knowledge on the subject. The understanding of the determinants of the living arrangements of the elderly can help in the planning of public policies that contribute to meet the health needs of this population and their families^7^. 

It is noteworthy that the determinants of the living arrangements differ according to the cultural, socioeconomic and social differences between countries^5^. In this way, this study allowed to bring the knowledge on these aspects to the Brazilian context, considering that this is a sample of the elderly population of a community of a municipality in the interior of Minas Gerais. However, living arrangements may vary between different regions of the same country^5^, which indicates the need of additional studies on the subject at a national level, in order to encourage the development of more specific health strategies to the Brazilian context.

This study has as possible limitation the sample loss during its follow-up. Despite this loss, the possible occurrence of selection bias is minimized because all the elderly who met the inclusion criteria during the investigation period were interviewed. 

Further investigations in larger scale are suggested, preferably multicentric, in order to verify the determinants of the living arrangements of the elderly, taking into account the sociodemographic, social and cultural characteristics of each context.

## Conclusion 

This study identified the characteristics of the living arrangements and their sociodemographic and health determinants. It was found that, among female elderly aged 80 or over, those who live alone accounted for the highest percentage during the follow-up. The number of morbidities increased in the three groups during the study, and the highest rates were observed in the elderly group who presented change in the dynamics of living arrangements. Sex and income were determining factors of the living arrangements, but the health variables were not associated with the outcome analyzed. 

It is believed that the findings of this study may contribute to the planning and management of health and social policies that aim to assist the elderly in different contexts in terms of the living arrangements. There is an urgent need to consider the most vulnerable elderly like those living alone, females, with older ages, who have lower incomes and chronic health conditions.

In the Family Health Strategy (FHS), nurses have the household context as privileged locus to check the care needs and the way in which the elderly and their families face difficulties in everyday life. To identify the most vulnerable groups, which need to be prioritized during home visits, nurses can make use of the available resources of the work process of the FHS, for example, the assessment of family risk. Other promising tools for the planning and implementation of health care are the genogram and eco-map that allow the view of the formal and informal resources available to the elderly and their family.
